# 

**Published:** 1852-04

**Authors:** 


### CONTENTS

### OF NO. IV.

---

### ORIGINAL COMMUNICATIONS.

### ESSAYS AND CASES.

Art. I.—A Clinical Lecture on Acute Pulmonary Phthisis. By David W. Yandell, M. D., one of the Consulting Physicians to the L. M. Hospital, of Louisville, Ky. 277
Art. II.—On the Use of Creasote in Diarrhea. By T. M. Woodson, M. D., of Sumner county, Tenn. - - - - 289
Art. III.—Disease, its Conditions and Treatment: An Inaugural Essay submitted to the Medical Faculty of the University of Louisville, March 5, 1852, for the degree of M. D. By Waldo Lewis, M. D., of Missouri. - 292

### REVIEWS.

Art. IV.—Discourses Delivered by appointment, before the Cincinnati Medical Library Association, January 9th and 10th, 1852. By Daniel Drake, M. D. - - - 303
Art. V.—Manual of Diseases of the Skin, from the French of M. M. Cazenave and Schedel, with notes and additions. By Thomas H. Buress, M. D., Surgeon to the Blenheim Street Dispensary for Diseases of the Skin, etc. Second American Edition, enlarged and corrected from the last French edition, with additional notes. By H. D. Bulkley, M. D., Physician of the New York Hospital; Fellow of the College of Physicians and Surgeons, New York: Lecturer on Diseases of the Skin, etc., etc. - 319
Art. VI.—Report of the Pennsylvania Hospital for the Insane for the year 1851. By Thomas S. Kirkbride, M. D., Physician to the Institution. - - - - - - 327

ART. VII.—Homœopathy: An Examination of its Doctrines and Evidences. By WORTHINGTON HOOKER, M. D., author of "Physician and Patient," and "Medical Delusions. - - - - - - - - 331

SELECTIONS FROM FOREIGN AND HOME JOURNALS.

A Case of Croup in which Tracheotomy was successfully employed. - - - - - - - - - 337
Case of Colica Pictonum, from the medical employment of Acetate of Lead. - - - 341
On the Effect of Chloroform on Muscular Fibre. - - - 346
On the Iodine of the Atmosphere. - - - - - - 347
Substitutes for Mercurials in the Treatment of Syphilis.- - - 348
On the Effects of Virulent Matters when taken into the Stomachs of Men and the Lower Animals. - - - - 349
Throat Diseases—Folliculitis. - - - - - - - 350
Ulceration and Perforation of the Stomach. - - - - 358

ORIGINAL INTELLIGENCE.

University of Louisville. - - - - - - - 361
Spiritual Writings. - - - - - - - - 362
Meteorological Table for the month of February, 1852. - - 368

